# GSMA: Gene Set Matrix Analysis, An Automated Method for Rapid Hypothesis Testing of Gene Expression Data

**Published:** 2009-11-24

**Authors:** Chris Cheadle, Tonya Watkins, Jinshui Fan, Marc A. Williams, Steven Georas, John Hall, Antony Rosen, Kathleen C. Barnes

**Affiliations:** 1 Genomics Core, Division of Allergy and Clinical Immunology, School of Medicine, Johns Hopkins University, 5200 Eastern Avenue, Baltimore, MD 21224; 2 University of Rochester School of Medicine and Dentistry, Division of Pulmonary and Critical Care Medicine, Rochester, New York, U.S.A; 3 Division of Rheumatology, School of Medicine, Johns Hopkins University, 5200 Eastern Avenue, Baltimore, MD 21224

## Abstract

**Background:**

Microarray technology has become highly valuable for identifying complex global changes in gene expression patterns. The assignment of functional information to these complex patterns remains a challenging task in effectively interpreting data and correlating results from across experiments, projects and laboratories. Methods which allow the rapid and robust evaluation of multiple functional hypotheses increase the power of individual researchers to data mine gene expression data more efficiently.

**Results:**

We have developed (gene set matrix analysis) GSMA as a useful method for the rapid testing of group-wise up- or down-regulation of gene expression simultaneously for multiple lists of genes (gene sets) against entire distributions of gene expression changes (datasets) for single or multiple experiments. The utility of GSMA lies in its flexibility to rapidly poll gene sets related by known biological function or as designated solely by the end-user against large numbers of datasets simultaneously.

**Conclusions:**

GSMA provides a simple and straightforward method for hypothesis testing in which genes are tested by groups across multiple datasets for patterns of expression enrichment.

## Background

Assigning functional meaning to patterns of statistically significant changes in gene expression is a common goal in the interpretation of microarray data. Until recently most conventional approaches have restricted their focus to only those genes which have satisfied multiple different criteria including size of fold change, significant p-value (often accompanied by additional requirements related to passing tests correcting for multiple comparisons), and certain minimum baseline levels of expression on at least one side of the comparison. This approach was reasonable during the early developmental period of microarrays when uncertainty as to the reliability of gene expression measurements naturally led to a conservative bias in the interpretation of microarray data in an effort to reduce, as much as possible, the inclusion of artifactual noise in analyses. Unfortunately, the tradeoff in reducing Type 1 error (false positives) was almost certainly at the expense of increasing Type II error (false negatives) but since these were essentially unknown, the problem tended to be ignored at that time. The issue has become more acute as technical improvements in microarray technology and the extent and depth of microarray studies have expanded at accelerated rates. The loss of vital information because of restrictive significance levels is less tolerable and, as others have argued, can result in the failure to define small but coordinated changes in gene expression which clearly, in the aggregate, distinguish biological phenotypes [1].

Traditional methods of assigning function to gene lists have focused primarily in looking for enrichment within a group of genes on the basis of some functional category, for example, for gene ontologies (GenMapp, David/Ease) or pathways (KEGG, BioCarta). These methods use some simple statistic (e.g. Fisher’s exact test) to generate an estimate of probability that the genes are enriched relative to all genes for that category and corrected for the frequency of representation for the genes of that category on the microarray platform being used. These methods are vulnerable to small changes in the genelist composition even among highly related experiments as a result of natural variation in the expression of genes close to preset significance thresholds. In addition, these methods tend to under-represent the population of truly regulated genes for a given category, again because of arbitrary significance thresholds, thus reducing the overall power of the analyses.

Recent, more promising developments in micro-array data analysis have succeeded where more traditional methods have failed [2] primarily as a result of inverting the analysis paradigm. Instead of examining a restricted list of genes selected by significance criteria for the enrichment of functionally related genes, these alternative methods take predetermined gene lists (or gene sets) often derived as described above (e.g. GO categories, pathways, common promoter elements) and use these gene sets to poll an entire dataset of gene expression changes. In this way, all the data is taken into consideration when computing enrichment statistics, and all the individual values of the particular difference metric used are taken into account. Gene sets derived from empirically determined gene expression signatures based solely on experimental data can also be used to interrogate additional datasets and demonstrate shared common patterns [3]. In fact, because of this unique ability to comprehensively compare gene expression results between experiments, we propose that these methods be referred to, in general, as gene expression signature analyses in order to distinguish them from the more conventional methods which consider only statistically significant genes as candidates for functional analysis [4–7].

Two variations of gene expression signature analyses have thus far been implemented, distinguished by, primarily, whether the position of the genes in a rank ordered dataset of gene expression differences is taken into account or not. The first major method to be described, gene set enrichment analysis (GSEA) is a non-parametric method in which the relative rank order of genes from a selected gene set is considered across the entire distribution of gene expression differences. This method essentially provides a weighting function which can identify subsets of genes within a gene list that are significantly enriched in a positive or negative direction (up- or down-regulated). Various versions of this approach have been reported [8, 9] including an alternative approach which can be used to detect the simultaneous significant enrichment of both up- and down-regulated genes within a single gene set [10].

In contrast to GSEA and the other related non-parametric methods, the parametric analysis of gene expression (PAGE) approach for gene expression signature analysis [11] involves the calculation of a single parameter (for example, the mean or median of expression difference values) for both the data extracted from a dataset by a particular gene list as well as for the dataset as a whole. The value of the gene list parameter is then compared with the same parameter derived for the entire dataset and statistically significant enrichment is indicated by a z score value (corrected for sample size). The parametric approach will not detect subsets of regulated genes contained within a given gene list because all of the gene list values are summarized in one aggregate parameter. Thus any distinction between sub-groups of genes within a given gene list is lost. On the other hand, a parametric approach such as PAGE is relatively easy to implement and, perhaps, even more importantly, as we will describe and demonstrate below, these parametric approaches are capable of being scaled-up and streamlined for rapid and very efficient high-throughput analysis of gene expression data.

## Implementation

GSMA is currently implemented in the JMP desktop statistical discovery software from SAS as a series of customized JMP scripts (supplementary information, file 6–8). Run times on a Windows XP platform, 1.0 GHZ, 512MB RAM, varies directly in proportion to the size and number of the datasets as well as the size of the genelist (large combinations, particularly of genelists >500, may require processing overnight, e.g. processing of the asthma related datasets versus pathways gene lists, as shown in Figure 3A, has a run time of little under one hour). GSMA can be performed either on single (one-dimensional 1D GSMA) or multiple datasets (two-dimensional 2D GSMA). The output between the two versions differs primarily on the data representations which are generated upon completion of the appropriate JMP scripts; all versions generate a file of GSMA z scores in tabular form. An additional variation available in both 1D and 2D GSMA is the substitution of median for mean calculations in order to reduce the influence of outliers on the computed GSMA z scores. 2D GSMA is available in a version 2 form which, in addition, to computing the basic z score matrix also captures the difference values for every gene in each list of a given gene set and automatically returns these results to an Excel workbook, using a separate worksheet for each list. Since this output is not practical to use for larger gene sets, it is usually incorporated into a second pass through the data with a subset of the original gene set tested for drill down purposes (for an example of the output of this process, see Fig. 3D below). GSMA scores are computed for each gene list for each dataset according to the algorithm first described by Kim and Volsky [11]:

$$\text{Z}=(\text{Sm}-\mu )*{\text{m}}^{1/2}/\sigma$$

Where Sm is the mean of the difference metric values of genes for a given gene list and the size of the given gene list is m. The mean (μ) and standard deviation (*σ*) of the total difference metric values for a given microarray dataset are calculated for all genes.

**Figure 1.**
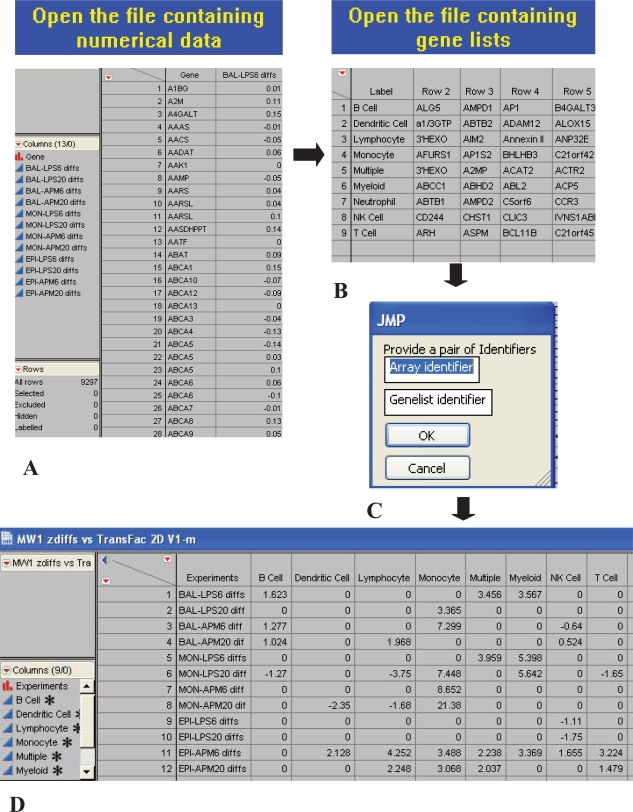
Screen shots of GSMA scripts implemented in JMP. Upon initiating the start-up of one of the GSMA scripts, the user is prompted to: **A.** up-load a tab-delimited file of gene expression differences, **B.** upload a tab delimited gene set query (list of lists), **C.** name the dataset and query lists, **D.** after the script finishes running, a matrix of z scores is returned for all lists in the gene set which exceed a pre-set significance threshold for any given dataset.

GSMA is initiated by the user by running the appropriate JMP script. The user will first be prompted to upload a tab-delimited file containing one or more columns of pre-computed changes in gene expression which can be in the form of simple differences between the means, fold-changes, log ratios, or any other consistent difference metric (Fig. 1). Each column of gene expression differences is referred to as a dataset. The first column in every file is devoted to gene IDs which are represented by the appropriate human gene symbols as accepted by the HUGO Gene Nomenclature Committee (mouse gene symbols must first be converted to their human gene symbol counterparts). The user can choose to use a different gene identifier (e.g. GenBank accession number) as long as care is taken to use the same identifier for both the datasets and gene lists. The script will next prompt the user to upload a query file which must contain at least one row of genes identified by HUGO gene symbols, as above, and again the first column contains the gene set name (there are no restrictions on the naming convention for gene lists in a given gene set). Finally, the user is asked to name both the dataset and gene set files. Submission of this information starts the GSMA protocols which are completed in a time proportional to the complexity of both the datasets and the query lists which are being tested (highly complex dataset/gene set combinations may require running the scripts overnight on most desktop PCs). A discussion of GSMA output and its interpretation will be the subject of the Results section below. The 2D GSMA script as well as sample GSMA datasets and a query gene list file are available from the supplementary information.

## Results

### 1D GSMA

**Figure 2.**
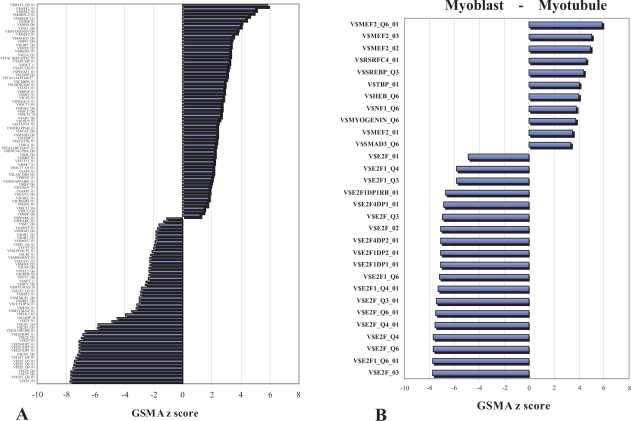
Graphical representation of GSMA significance scores for **A.** 114/445 genelists derived from the Transfac database [13] rank ordered from high to low on the basis of the GSMA z scores, and **B.** 30 of the most significant TransFac gene lists which correspond to gene groups whose overall expression was either increased during myotube formation (positive z scores) or increased in myoblasts (negative z scores).

The simplest instance of GSMA (one-dimensional or 1D GSMA) tests one dataset (one column of data) of gene expression differences against a given gene set. Figure 2A shows an example of this form of GSMA in which GSMA z scores returns have been rank ordered and presented in the form of a bar graph with the largest positive z score at the top and the largest negative z score at the bottom. This axis of values corresponds to increasing positive or negative enrichment of gene expression for the genes in these lists as calculated between treatment and control. For this example, gene expression differences were calculated by taking the average of samples pre- and post-induction (by serum withdrawal) in a human model of myocyte differentiation of proliferating cells (myoblasts) to mature myotubes [12]. In order to simplify the analysis a series of highly replicated time course samples were collapsed into 2 groups for comparison.

It should be noted that gene sets are themselves lists of lists, as in this case, a compendium of 445 separate gene lists (gene symbols only) was tested. Each list is composed of a variable number of genes grouped by having a particular transcription factor binding site in its upstream promoter region (Trans-Fac gene set) [13]. Gene set lists may and often do have gene redundancies, ie,the same gene may appear in many different lists within a single gene set as, for example, does the JUND gene which is found in a total of 29 lists within the TransFac gene set. Each instance of JUND in a TransFac list corresponds to a different transcription factor binding site mapped within the JUND gene promoter. While the individual contribution of redundant genes is always the same for a particular dataset, the output from the various lists in which they are located is context-dependent and can be highly variable.

Figure 2B shows the thirty most highly enriched GSMA gene lists from the TransFac gene set representing groups of genes whose overall expression has either increased during myotube formation or whose expression is more dominant at the myoblast stage. Gene lists corresponding to the E2F family of transcription factor binding sites are highly enriched in the myoblast direction relative to differentiated myo-tube cells. E2F proteins are known to play a key role in the expression of genes required for the movement into and through the cell cycle progression and thus their transcription is emphasized in the rapidly dividing myoblast cells. Gene lists known to be related to myogenesis, on the other hand, are enriched in the myotube axis of differentiation including genes controlled by myocyte enhancer factor 2 (**MEF2**) a class of transcription factors essential for muscle development, myogenin which is required not for the initiation of myogenesis but instead for skeletal muscle formation [14], **RSRFC4** which recognizes similar but distinct binding sites found in the promoters of both muscle-specific and ‘immediate early’ genes [15]. **HEB**, a helix-loop-helix protein, can modulate the DNA-binding ability of myogenic regulatory factors (MRFs) [16–18]. Isoforms of **NFI** proteins accumulate differentially in fast- and slow-twitch muscles and are thought to contribute to the molecular basis for skeletal muscle diversity [19]. The up-regulation of genes associated with **SREPB** (sterol response element binding protein) is somewhat of a puzzle as this transcription factor is strongly associated with adipocyte determination and differentiation [20]. Both adipocytes and myocytes can be induced from the same multi-potent mesodermal progenitor cell type [21] depending on the conditions used. It has been well established that peroxisome proliferator-activated receptor γ (PPARγ) is an absolute requirement for adipocyte differentiation and although some statistically significant up-regulation of expression of PPARγ was observed during myotube formation in these experiments, overall it remained at relatively low levels (data not shown). It is intriguing to speculate that perhaps SREBP is generally up-regulated during differentiation in this cell type but only induces adipogenesis in the presence of a correspondingly robust increase of PPARγ.

### 2D GSMA

**Figure 3.**
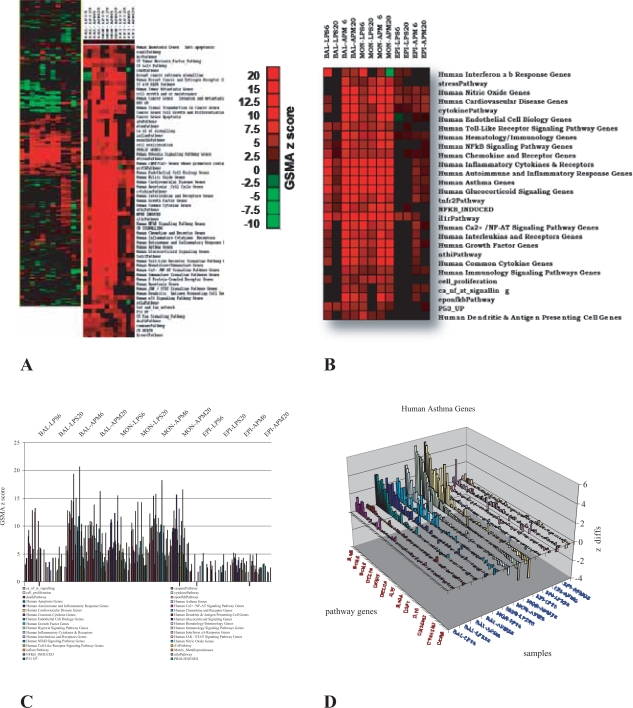
2D GSMA results corresponding to 529 pathway-related gene lists tested independently on 12 separate datasets. **A.** Thumbnail image—heatmap of hierarchical clustering of GSMA z scores. **B.** Zoom image—highlighting an area of extensive pathway co-regulation shared by many but not all samples. **C.** GSMA z score data from 3B displayed in column format. **D.** Example of gene expression differences from a single enriched pathway (human asthma genes) for all samples. Samples were derived from multiple patients and stimulated in culture with lipopolysaccharide (LPS) or ambient particulate matter (APM) for 6 and 20 hours. BAL = bronchial lavage macrophages, MON = monocytes, EPI = airway epithelial cells.

It is as of much value to investigate patterns of systematic enrichment within and between multiple experiments by clustering gene sets (Fig. 3) as it is to look for patterns of coordinate gene expression at the individual gene level. The use of gene expression signature analysis in this way has been previously suggested by others [9]. GSMA provides a straightforward and highly scalable method for analyzing very large combinations of datasets, gene sets, and the resulting GSMA values in a simple and efficient manner. Figure 3 shows a composite of results of 2D GSMA analysis in which a pathway gene set containing 587 separate genelists was tested against 12 separate datasets derived from 3 cell types activated with 2 different antigenic stimuli (LPS and ambient particulate matter—APM [22]). Patterns of common pathway enrichment are clearly visible by clustering (unsupervised, single linkage, hierarchical clustering using uncentered Pearson correlations [23]), the GSMA z score matrix and generating a heat map of the results (Fig. 3A). An overlapping and robust response to antigenic stimuli is demonstrated in both bronchiolar lavage macrophages (BAL) and peripheral blood monocytes (MON) but not in airway epithelium (Fig. 3B). The strongest interferon response in both immune cell types is induced by LPS at 6 hours. Overall response patterns to LPS treatment are subsiding in bronchial macrophages but continue to be prolonged in circulating monocytes after 20 hours post-induction. Graphical representation of the same data (Fig. 3C) emphasizes the relatively minor response of airway epithelial cells and the differential response of macrophages and monocytes to LPS and APM in terms of the magnitude of pathway involvement. The high granularity of GSMA data is demonstrated by patterns of individual gene enrichment within a single example of a positively regulated gene list (Fig. 3D). The pattern of human asthma-related genes, on a gene-by-gene basis, almost exactly maps to the patterns exhibited at the gene list level and, in addition, provides the end-user immediate access to the basic microarray data measurements—changes in expression at the individual gene evel. These genes are now conveniently organized by both function and experiment for further consideration.

### GSMA and hypothesis evaluation

The reliability and usefulness of a newly introduced data mining tool is often (and understandably) evaluated by its ability to return information which is already well understood, previously documented, and accepted in other contexts. So, for example, in the clustering data in Figure 3 above, the large overlap between stimulated bronchial macrophages and peripheral blood monocytes for pathways involved in the immune response was not surprising in the least, and neither was the relative absence of enrichment of these same pathways for airway epithelial cells subjected to the same treatments. These results are in fact, offered simply as a proof of principle that GSMA can produce logical and coherent outcomes given a large number of datasets and gene set gene lists. The next step for demonstrating the usefulness of a data-mining tool is to show evidence for the discovery of new knowledge. The following two cases are offered as examples of how GSMA can add value by systematically exploring relationships contained within gene expression data.

#### The case of the non-responsive patient

**Figure 4.**
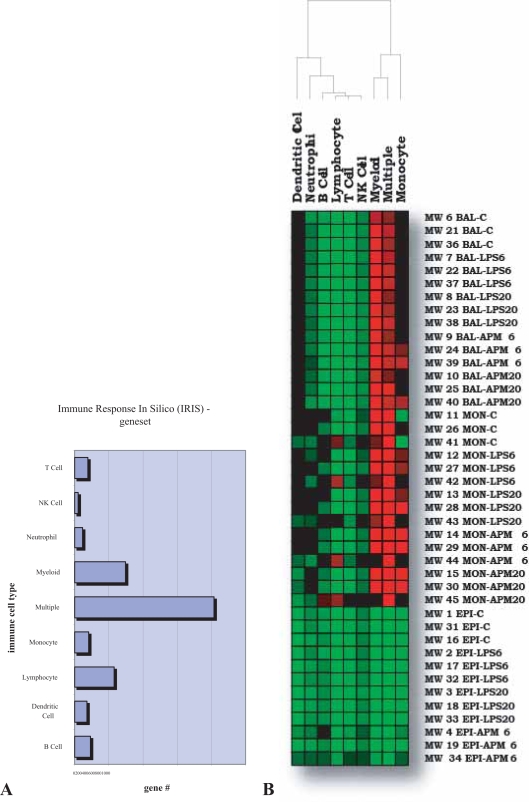
Application of GSMA directly to gene expression intensities. **A.** Bar graph illustrating the distribution of the numbers of specific immuno-dominant genes compiled from a comprehensive compendium of microarray human gene expression data from six key immune cell types [25]. **B.** Hierarchical clustering of GSMA results in which the average gene expression intensities of the various cell types and treatments tested (vertical axis) were evaluated using the IRIS gene set and assigned a z score value on the basis of enrichment for the immune cell types as shown (horizontal axis). GSMA results for this visualization were clustered by IRIS immune cell type while the experimental cell type (BAL, MON, & EPI) and the treatment (LPS & APM) order was held constant.

The use of GSMA is not restricted to testing data-sets of changes in gene expression but can also be applied directly to gene expression intensity data as well. When the z transformation method is used for gene expression normalization [24], gene expression data is distributed evenly above and below zero in log_10_ space. When GSMA is applied directly to gene expression intensities, the average overall intensity for a given list of genes is translated directly into a corresponding GSMA z score with a high GSMA score (red) indicating that the genes for that particular list were highly expressed on average. Conversely, a low GSMA score (green) indicates an overall low level of gene expression for a given gene list. As previously mentioned, one of the advantages of GSMA is that gene lists can be derived from virtually any source and can be quickly and easily converted into testable gene sets. In the following example, good advantage was taken of the public on-line availability of lists of genes related to immune cell-specific expression developed from experimental data by researchers at Genentech Inc. [25]. Figure 4A shows the gene numbers by list type contained within the IRIS (Immune Response *In Silico*) gene set. Bronchial lavage macrophages and peripheral blood monocytes are easily distinguished from airway epithelial cells by virtue of their dramatically different expression of immune cell specific genes in Figure 4B. Macrophage and monocyte cells are, not surprisingly, enriched for the expression of genes associated with myeloid, monocyte and multiple immune cell types while airway epithelial show no strong enrichment for any group of immune related genes. Interestingly, monocyte-specific genes are induced in both BAL and MON cells with antigenic stimuli but otherwise they remain at moderate or low levels in resting cells. As expected, the monocyte specific marker genes are mobilized as a group in a more robust fashion in monocytes than macrophages for both LPS and APM at the time points tested. A closer examination of the GSMA scores for the monocytes and the monocyte-specific gene list reveals a noteworthy anomaly. One patient (41, 42, … 45) appears not to be generating any strong monocyte-specific gene expression signature response to antigen in cultured monocytes while at the same time this cell population is instead distinctly and uniquely enriched for lymphocyte-specific gene expression. The BAL cells for the same patient (36, 37, … 40) are the primary contributors to a monocyte-specific gene expression response in BAL cells overall. It is tempting to speculate that this patient may have been undergoing an active inflammatory response at the time of cell harvest. It is also possible (but less likely) that this data could be the result of some variation in the collection procedure. What is clear, however, is that this patient is showing a differential response pattern which otherwise would have gone undetected without GSMA analysis and the inclusion of this data could adversely affect both the power and statistics of the study in question.

#### An hypothesis overturned

Recent work by ourselves and others has demonstrated widespread patterns of mRNA decay rate regulation in response to different biological stimuli [26–28]. The data supporting these conclusions has been generated primarily through direct comparison of nascent gene transcription (nuclear run-on RNA; NRO RNA) to changes in gene expression as measured at the whole cell level (Total RNA). Although these results have been validated by Actinomycin D chase assays on nuclear run-on RNA for selected genes [28], the vast majority of these observations are made by inference, through the recording of large and consistent changes in gene expression as measured in total RNA without a concomitant change in gene expression in the NRO RNA even when allowing for a time lag for mRNA processing and transport. These studies taken together suggest that genome wide post-transcriptional regulation of cellular mRNA levels is a widespread phenomenon and can account for as much as 50% of the changes in gene expression as measured by conventional microarray.

**Figure 5.**
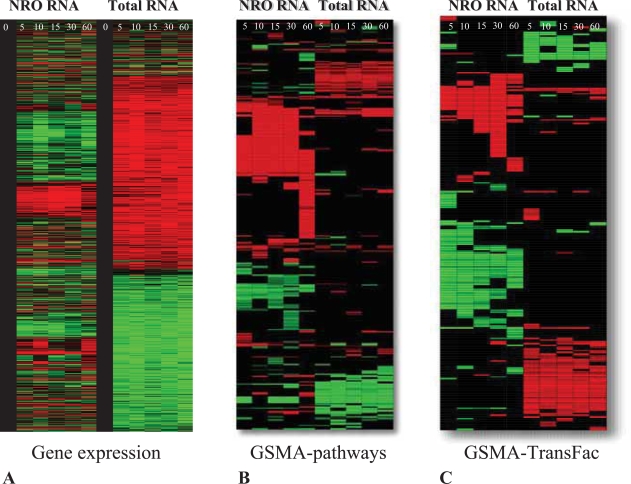
Characterization of contrasting nuclear run-on (NRO) and total RNA gene expression during a one hour time course (in minutes as indicated) of T cell activation. **A.** Heat map of hierarchical clustering of differential gene expression of NRO and total RNA up- (red) or down- (green) regulated from their respective baselines. **B.** 2D GSMA pathway results using the same dataset as in **5A** for analysis. **C.** 2D GSMA transcription factor binding site (TFBS - TransFac) results using the same dataset as in **5A** for analysis.

An example of this type of study is seen in Figure 5A in which changes in gene expression are contrasted between NRO and Total RNA across a one hour time course of T cell activation. Consistent and overlapping regulation of gene expression across the entire length of the time course can be seen for both NRO and Total RNA. Even greater numbers of cellular mRNAs, however, are clearly being regulated while relatively low or no regulation can be detected for the same genes in the NRO RNA. Initially our working hypothesis was that the demonstrably large numbers of post-transcriptional changes in gene expression would explain why it has been difficult to routinely correlate changes in total cellular gene expression with common upstream promoter elements in organisms higher than yeast [29]. For example, in our own work, we were able to demonstrate the enrichment of genes containing either the NFKB or NFAT families of transcription factor binding sites during the activation of Jurkat T cells in NRO but not total RNA [30].

**Figure 6.**
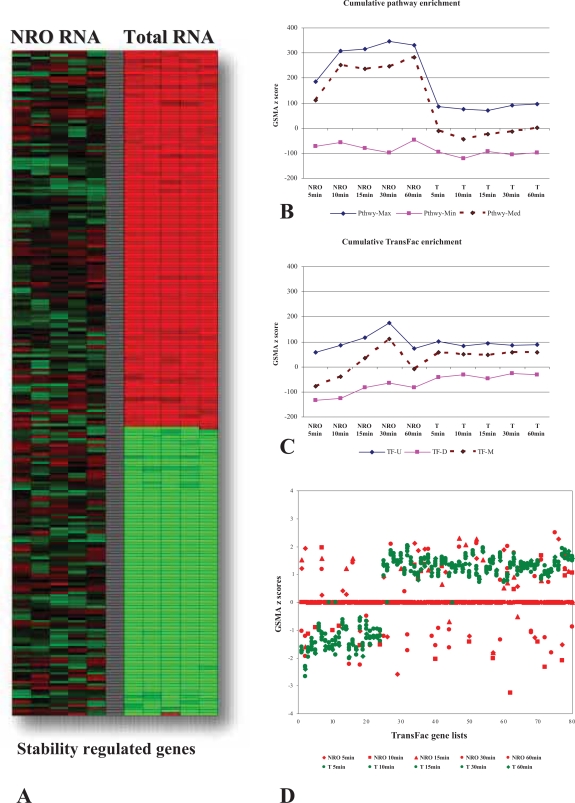
Characterization of pathway and TFBS gene set enrichment between nuclear run-on (NRO) and total RNA during a time course of T cell activation. **A.** Subset of stability regulated genes. **B.** Comparison of cumulative pathway enrichment between nuclear-run on (NRO) and total RNA during T cell activation. **C.** Comparison of cumulative transcription factor binding site (TransFac) enrichment between nuclear-run on (NRO) and total RNA during T cell activation. In both 6B & 6C above; -U = the sum of all positively enriched gene lists, -D = the sum of all negatively enriched gene lists, -M = the sum of all enriched pathways. **D.** Specific TFBS (TransFac) gene set enrichment in total but not NRO RNA for a selected set of stability regulated genes.

GSMA analysis of this dataset (Fig. 5B) shows the distinctive and contrasting patterns of pathway enrichment between the NRO and Total RNA measurements during Jurkat T cell activation. NRO gene transcription heavily favors the up-regulation of specific pathways (in other words, turning on gene expression occurs more frequently than turning it off) while pathway enrichments for total RNA are approximately evenly divided between up- and down-regulation (Fig. 6B). The NRO pattern of pathway enrichment, in particular, shows a dramatic shift of emphasis between 30 and 60 minutes of activation including the turning on of genes in NFKB pathways (supplementary data) as predicted by our previous findings [30].

Similarly, when GSMA was performed on the same datasets with the TransFac gene lists, the NRO and total RNA patterns of enrichment were again quite different (Fig. 5C). The coordinated increase in the expression of genes correlated by TransFac gene lists appears to gather momentum up to a spike at 30 minutes in NRO RNA while in the total RNA there is a slight but consistent bias towards up-regulation of TransFac gene lists across the time course of activation (Fig. 6C). The appearance of substantial regulation correlated by the TransFac gene lists in total RNA was surprising given that many of these genes we believe to be primarily regulated by changes in mRNA decay rates and therefore we did not expect to detect a particularly large association with transcriptional control elements in this analysis. In order to focus directly on the question of whether or not stability regulated genes somehow continue to maintain a functional association correlated to the control of their transcription, a subset of the total RNA genes selected as most likely to be stability regulated genes (Fig. 6A) was tested again with the TransFac genelists and the results (Fig. 6D) showed substantial up and down regulation of Total RNA genes correlated by the presence of common transcription factor binding sites while the corresponding NRO data generated little or no GSMA z score values. It became clear via this analysis that there exists a good possibility that the functional characteristics predicted among genes by the presence of common upstream promoter elements (e.g. transcription factor binding sites; TFBS) can be found not only in the coordinated expression of nascent gene transcription but also carries over to guide at least a portion of post transcriptional regulation as well. In other words, the functional associations which result in coordinated gene expression at the transcriptional level continue to have relevance in later whole cell regulation of gene expression in which, presumably, the presence or absence of a TFBS no longer has a direct mechanistic relevance. This finding directly contradicted our previous assumptions and was only suggested and revealed by GSMA analysis of patterns of shared TFBS in these datasets.

## Discussion

The key to effective data-mining of gene expression data rests not only in the kinds of questions asked but also the ease and efficiency which the answers can be obtained. GSMA offers a simple but effective tool for rapidly exploring specific patterns of gene enrichment by groups across a very wide range of experiments. GSMA has a simple user-friendly interface (Fig. 1), running scripts in JMP, an inexpensive and widely available windows version of SAS. The end-user supplies an input of gene expression data annotated with HUGO gene symbols and a second input of gene lists similarly annotated. The user has the option (script dependent) for a 1D (Fig. 2) or 2D (Fig. 3) analysis, as well as specifying whether to use the mean or median in calculating the gene enrichment scores. In addition, there is also a GSMA 2D version which will return not only the matrix of GSMA z scores but will also isolate the input expression data for each gene list of a given gene set, each directly to a separate Excel worksheet (an example of a chart constructed directly from the data contained on one of these sheets is shown in Fig. 3D). We find that this option works best for a limited number of genelists (10–20) simply in terms of practicability and is particularly useful for drill-down purposes.

GSMA can be used for comparing changes in gene expression between many different experiments or for testing for group-wise changing patterns of normalized gene expression intensities directly. Examination of gene expression intensities using a gene set specific for immune cell expression dominance clearly revealed an anomalous response from one of the patients tested in the asthma dataset (Fig. 4A). This discovery is both of clinical relevance to the study being conducted as well as illustrating the flexibility of GSMA for visualizing complex datasets in a creative and easily customized fashion.

Finally, in Figures 5 and 6, we provide an example of how GSMA was used to discover unexpected patterns of TFBS enrichment in the upstream promoter regions of genes considered to be primarily post-transcriptionally regulated. This finding was only made possible by the ease with which GSMA can be used to interrogate gene expression data comprehensively using very large gene sets (the TransFac gene set contains 445 separate gene lists), a task that just a short time ago would have been prohibitive for a moderately sized laboratory solely for the purposes of exploration. It was unclear initially whether or not searching gene expression data by individual TFBS would even be appropriate given the full complexity of promoter architecture [31, 32]. It is now becoming apparent that there are indeed strong correlations to be found between generalized gene expression and the presence of individual common promoter elements as found by ourselves and others [33].

## Conclusion

As the amount of gene expression data expands exponentially and the volume of stored data available in repositories both public and private surges, it becomes increasingly important to develop methods which can accelerate the process of hypothesis testing at multiple levels of either the individual experiment, across projects, and even within an entire database [34]. GSMA and similar approaches will be useful for rapidly testing original as well as archived gene expression datasets for specific gene expression enrichment at the group level using either preset gene lists (pathways, promoter elements, disease association, etc) or empirically derived gene expression signatures [2]. These methods should be sufficiently permissive so as to allow for the natural variation inherent to biological systems while at the same time be sufficiently quantitative to facilitate the prioritizing of new knowledge and help to organize it in a coherent way.

## Abbreviations

BAL: bronchial alveolar lavage macrophage;
GSMA: gene set matrix analysis;
MON: peripheral blood monocyte;
NRO: nuclear run-on;
TFBS: transcription factor binding sites.

## Additional files

Additional file 1. Excel workbook, Cheadle et al.– 1D GSMA myotube differentiation versus TransFac gene lists - z score matrix for Figure 2.

Additional file 2. Excel workbook, Cheadle et al. – 2D GSMA asthma dataset versus Pathways gene lists - z score matrix for Figure 3, also gene specific difference values for the human asthma genes for Figure 3D.

Additional file 3. Excel workbook, Cheadle et al. – 2D GSMA asthma dataset versus IRIS gene lists - z score matrix for Figure 4.

Additional file 4. Excel workbook, Cheadle et al. – 2D GSMA T cell activation, NRO and total RNA versus Pathways and TransFac gene lists - z score matrix for Figure 5 (data for all NRO and total RNA gene expression differences (Fig. 5A) available at [30]).

Additional file 5. Excel workbook, Cheadle et al. – Stability-regulated genes.

Additional file 6. JMP script, Cheadle et al. – 2D GSMA, medians, all values reported.

Additional file 7. TAB text file, Cheadle et al. – test dataset (w/files 6 and 8, will generate data for Fig. 4B.)

Additional file 8. JMP file, Cheadle et al. – test genelist (w/files 6 and 7, will generate data for Fig. 4B.)
